# Prospective surveillance of women with a family history of breast cancer: auditing the risk threshold

**DOI:** 10.1038/sj.bjc.6604155

**Published:** 2008-02-19

**Authors:** E Anderson, J Berg, R Black, N Bradshaw, J Campbell, H Carnaghan, R Cetnarkyj, S Drummond, R Davidson, J Dunlop, A Fordyce, B Gibbons, D Goudie, H Gregory, S Holloway, M Longmuir, L McLeish, V Murday, Z Miedzybrodska, D Nicholson, P Pearson, M Porteous, M Reis, S Slater, K Smith, E Smyth, L Snadden, M Steel, D Stirling, C Watt, C Whyte, D Young

**Affiliations:** 1 Edinburgh Breast Cancer Family Service, Department of Clinical Genetics and Edinburgh Breast Unit, Western General Hospital, Edinburgh EH4 2XU, UK; 2 Dundee Breast Cancer Family Service, Departments of Clinical Genetics and Breast Unit, Ninewells Hospital, Dundee DD1 9SY, UK; 3 NHS Scotland Information Services Division, Epidemiology and Statistics Group and Scottish Cancer Registry, Gyle Square, Edinburgh EH12 9EB, UK; 4 Glasgow Breast Cancer Family Service, Ferguson Smith Centre for Clinical Genetics, Yorkhill Hospitals, Glasgow G3 8SJ, UK; 5 Bute Medical School, University of St Andrews, St Andrews KY16 9TS, UK; 6 Aberdeen Breast Cancer Family Service, Department of Medical Genetics, Aberdeen Royal Infirmary, Aberdeen AB25 2ZD, UK

**Keywords:** breast cancer, familial, risk assessment

## Abstract

To evaluate current guidelines criteria for inclusion of women in special ‘breast cancer family history’ surveillance programmes, records were reviewed of women referred to Scottish breast cancer family clinics between January 1994 and December 2003 but discharged as at ‘less than ‘moderate’ familial risk’. The Scottish Cancer Registry was then interrogated to determine subsequent age-specific incidence of breast cancer in this cohort and corresponding Scottish population figures. Among 2074 women, with an average follow-up of 4.0 years, 28 invasive breast cancers were recorded up to December 2003, where 14.4 were expected, a relative risk (RR) of 1.94. Eleven further breast cancers were recorded between January 2004 and February 2006 (ascertainment incomplete for this period). The overall RR for women in the study cohort exceeded the accepted ‘cutoff’ level (RR=1.7) for provision of special counselling and surveillance. The highest RR was found for the age group 45–59 years and this group also generated the majority of breast cancers. The National Institute for Clinical Excellence (‘NICE’) guidelines appear to be more accurate than those of the Scottish Intercollegiate Guidelines Network (‘SIGN’) in defining ‘moderate’ familial risk, and longer follow-up of this cohort could generate an evidence base for further modification of familial breast cancer services.

A family history of breast cancer is recognised as a risk factor for the disease. Most efforts to quantify that risk rely upon two retrospective studies (Claus *et al*, 1991; Collaborative Group on Hormonal Factors in Breast Cancer, 2001), both of which provide a range of estimates, based on the number of affected close relatives and their ages at diagnosis. These estimates, however, are derived from rather small numbers of women in some of the specific risk categories and have wide confidence intervals. The National Institute for Clinical Excellence (NICE) (2004) guideline on familial breast cancer concludes that ‘validation of risk assessment models is urgently needed’. It is universally accepted that a woman under 50 years who has three or more close relatives affected with breast cancer (one at least being a first-degree relative or second degree if on her father's side of the family) is at sufficiently increased risk to justify special surveillance, with the object of detecting breast cancer early in women too young to be included in the National Breast Screening Programme (NBSP). If there are only two close relatives affected, SIGN guidelines stipulate that both must have been under 60 years at diagnosis, but NICE (both the 2004 version and the 2006 update) applies no such age restriction. Under either NICE or SIGN criteria, if there has been only a single first-degree relative affected, risk to sister or daughter is not considered to be significantly increased unless age at diagnosis was less than 40 years. Both guidelines also take account of ovarian cancer among relatives, attaching slightly greater weight to it than to breast cancer in assessing familial risk. A prospective evaluation of five different protocols for prediction of breast cancer risk from family history and other criteria (Amir *et al*, 2003) concluded that the most commonly applied models tended to underestimate risk, particularly for those at the lower end of the familial risk spectrum but, again, numbers were limited. We have therefore embarked on a prospective survey of cancer incidence among a large cohort of asymptomatic women discharged from breast cancer family clinics because their risk levels, based on reported family histories, were judged insufficient to warrant enrolment in special surveillance programmes.

## PATIENTS AND METHODS

Since 1994, clinical services for familial breast cancer have been provided for the whole of Scotland by a network comprising four major centres in Aberdeen, Dundee, Edinburgh and Glasgow, involving close collaboration between medical genetics, diagnostic radiology and breast surgery units, with support from the Information Services Division (ISD) of the NHS Scotland Common Services Agency and the Scottish Cancer Registry. Two of the centres operate ‘one-stop’ clinics staffed by geneticists, breast surgeons and radiologists. The others provide comparable, but sequential, services in separate genetics and breast surgery clinics. In all four centres practice is based on guidelines formulated by the Scottish Office Home and Health Department (1998) and incorporated into Scottish Intercollegiate guidelines Network (SIGN) Guideline 29, 1998. These set a threshold, for the definition of ‘moderate’ familial risk, the major criteria being summarised above. Women referred to a breast cancer family service whose family histories meet or exceed these criteria are offered regular surveillance from age 35 (or 5 years younger than the earliest age of disease onset in a relative). Otherwise, they are discharged to primary care with reassurance, advice on being ‘breast aware’, encouragement to take advantage of the National Breast Screening Programme (NBSP) from age of 50 and a request to notify the cancer genetics service should any new breast or ovarian cancers occur within the family. The threshold requirements are in line with those proposed in the majority of published recommendations (Public Health Genetics Unit, 1998; Watson *et al*, 1999; Eccles *et al*, 2000; Haites *et al*, 2002; Breakthrough Breast Cancer, 2004; Nelson *et al*, 2005) but differ from NICE guidelines which remove any age-at-diagnosis restriction in the case of two affected relatives.

With local approval for audit purposes, the records of the four Scottish breast cancer family services were scrutinised to identify all referred women whose risk had been assessed, as below the ‘moderate’ threshold over the 10-year period from January 1994 to December 2003. Then, with consent from the Privacy Committee, Scottish Cancer Registry records were checked to detect any cancers recorded in this cohort of individuals. For breast and ovarian cancers, confirmation and further pathological details were sought from case notes.

For each woman referred to a cancer family clinic, but not offered continuing surveillance, the period elapsing between discharge from the service and December 2003 was calculated and, from that and the date of birth, the number of woman years of observation within one or more 5-year age spans (35–39, 40–44 and so on) was derived. The data were aggregated to give the total number of woman years of observation per 5-year age group and the corresponding ‘expected’ numbers of breast cancers were obtained from Scottish Cancer Registry figures, which report annual incidence for the same age groups. Where a breast or ovarian cancer had been recorded, the precise details of family history were re-checked in every case from cancer family clinic records. Cancers registered after December 2003 were noted and checked as above. Because registration is still incomplete for that period, these cases cannot be included in calculations of absolute or relative age-specific incidence but can be added to the earlier cases to record age distribution of cancers, their clinical and pathological characteristics and the proportions that would have met NICE criteria for inclusion in special surveillance programmes.

## RESULTS

**Figure 1:**
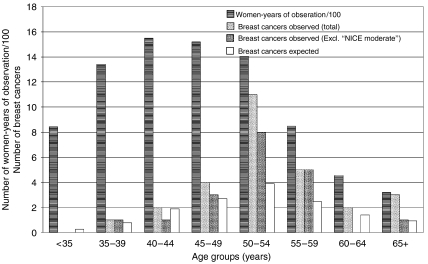
Age-group distribution of 28 invasive breast cancers recorded by the Scottish Cancer Registry from January 1994 to December 2003 among 2074 women discharged from the Scottish Breast Cancer Family clinics as being at less than ‘threshold’ level of risk: the same data but excluding cancers occurring among women whose risk would have been above the threshold under 2004 NICE guidelines (‘NICE moderate’ group) and expected distribution of breast cancers among 2074 unselected Scottish women with the same age distribution.

**Figure 2:**
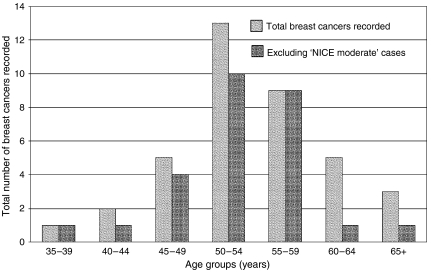
Age distribution of 39 invasive breast cancers recorded up to February 2006 in the study cohort (records incomplete beyond December 2003 so comparison with expected numbers would be invalid).

The principal findings are summarised in Table 1a and b and Figure 1. Within the main 10-year study period, 28 invasive breast cancers were recorded in 26 women out of the total cohort of 2074. The expected number was 14.4, giving an overall relative risk (RR) of 1.94 (95% CI=1.3–2.8). As shown in Figure 1, the RR was not uniform for all age groups, the highest (>2.8) being for the 50–54 year olds. The 11 further breast cancers diagnosed since December 2003 are listed in Table 1b and included in Figure 2.

Two women had suffered synchronous bilateral breast cancers, in each case both tumours being detected at first NBSP mammogram at the age of 50 years. One had a family history that would have placed her in the ‘NICE moderate’ risk category (sister diagnosed at the age of 46 years, paternal grandmother in her 70's). The other had only one affected relative, her mother, diagnosed at the age of 70 years. She would therefore have been considered below threshold risk level for surveillance under any extant guidelines.

In addition to the breast cancers recorded in Table 1a and b, there were three instances of ductal carcinoma *in situ* (DCIS) and one patient in whom an unsuspected second focus of invasive ductal cancer was identified by the pathologist in the mastectomy specimen following surgery for a symptomatic cancer. Although this was considered a second primary, that could not be proved and it has not been treated as such in the present report. One additional case of DCIS was recorded in 2005 and has been excluded from calculations. More than half of all breast cancers were diagnosed at clinical or mammographic screening – mainly through the NBSP, but three at one of the multi-disciplinary breast cancer family clinics, where policy (until the year 2004) had been to see all women referred, even where their risk had been evaluated and the decision already taken to discharge them following that single visit. Since 2004, women whose risk has been assessed as below the ‘moderate’ threshold are no longer seen at the multi-disciplinary clinic and are not offered clinical examination or a mammogram before discharge.

Twelve of the 39 tumours (31%) occurred in women whose family histories would have placed them in the ‘moderate’ risk category had NICE, rather than SIGN guidelines been applied – that is, they had two affected close relatives (one first degree) at least one of whom had been over 60 at diagnosis. A re-examination of the family histories of our total cohort shows that only 10% would have been reclassified as ‘moderate risk’ under NICE criteria (‘NICE moderate’ subgroup).

There were three epithelial ovarian cancers (one each of serous, mucinous and endometrioid type) and one borderline ovarian tumour. Only two ovarian cancers would have been expected but numbers are too small to draw any inference as yet.

## DISCUSSION

The overall RR found in this study approached 2.0, appreciably higher than the level of 1.7, which NICE and most other guidelines accept as the threshold above which women should be offered enrolment in a surveillance programme. Given that Cancer Registry data can never be 100% complete since, for example, any cancers occurring in members of our study cohort who had left Scotland would not be recorded, the RR we have calculated is a conservative figure. Furthermore, it is consistent with the findings of the very large reanalysis of epidemiological studies on familial breast cancer (Collaborative Group on Hormonal Factors in Breast Cancer, 2001), which cites a RR of almost two for women with one relative affected between the ages of 40 and 54 years. It is also in keeping with the conclusion of Amir *et al* (2003) that most currently applied algorithms underestimate the RRs associated with ‘weak’ family histories.

When NICE criteria are applied, 9 of the 28 cancers recorded before the end of the year 2003 were in women at ‘moderate’ risk. This applies to 12 cancers (in 11 women) from the total series of 39 (30.8%). These potentially ‘moderate risk’ women thus appear to be over-represented among those who subsequently developed breast cancer, relative to their numbers in the study cohort. If they are excluded, the overall RR falls to 1.32 (95% CI=0.8–2.1). However, 6 of the 11 NICE ‘moderate risk’ group were 60 or over at discharge from the cancer family service, whereas women of that age represented less than 10% of our total study population. Elderly people will, of course, have more elderly close relatives and, as breast cancer is an age-related disease, they may have more affected relatives without necessarily implying an increased familial risk.

Different issues arise in relation to the other end of the age spectrum. Where the family history is not strong, the chances of a major gene mutation (BRCA1 or BRCA2) being present are small and so too is the risk of very early-onset breast cancer. The RR in our cohort for women up to the age of 44 years is only 1.05, even if the single ‘NICE moderate’ patient is included. This age group comprised 45% of our total cohort and, while little weight can be attached to just three cancers, the findings suggest that NICE referral criteria are satisfactory for women under the age of 45 years, that is, the incidence of breast cancer among those who do not ‘qualify’ for special surveillance through a cancer family clinic is no higher than expected for the general population.

Our findings are most relevant to women aged 45–59 years. They accounted for 46% of our total woman years of follow-up but generated 71% of the breast cancers (84% if the NICE ‘moderate risk’ cases are excluded), and had a RR of 2.2 (95% CI=1.4–3.4) or 1.79 (95% CI=1.00–2.85) with the exclusion. Given that all but 3 of these 27 cancers had actually been diagnosed by the age of 56 years, there is a reasonable expectation that most would have been screen detected in a surveillance programme that provided regular mammography upto the age of 55 years. In fact, 10 of the 13 invasive breast cancers (77%) diagnosed in women from this cohort between the ages of 50 and 52 years were screen-detected, which contrasts with the corresponding figure from ISD of only 41% for the same age group in the unselected Scottish population (*P*<0.02), suggesting that women who had been discharged from a breast cancer family clinic were particularly motivated to attend for breast screening from the age of 50 years and/or that their tumours were slow growing and hence more amenable to screen detection. NICE criteria include a stipulation that ‘moderate’ risk means at least a 3% absolute risk of breast cancer between the ages of 40 and 50 years. Restricting analysis to the 1994–2003 cohort, we find that, even excluding the ‘NICE moderate’ cases, and assuming that half of the cancers detected at the age of 50 or 51 years would have been diagnosed by the age of 50 years in an appropriate screening programme, the cumulative risk over the 5 years from the age of 45 years was 2.3%, whereas the corresponding figure for the 10-year age span 45–55 (with the same assumption that half the cancers diagnosed at the age of 56 years might have been detected by the age of 55 years through screening) is 4.8%. Overall, these findings suggest that, for women aged 45–55 years, family history criteria for inclusion in breast cancer surveillance programmes should be kept under review.

The effect of the breast cancers diagnosed after discharge from the family history clinics would have been to raise the estimated familial risk level for close relatives, in many cases making them eligible for inclusion in regular surveillance programmes. In two instances, where the original family history was of one first-degree relative diagnosed over the age of 40 years, the onset of breast cancer in our patient was followed within 1 year by the same diagnosis in a sister, also at an early age, transforming both families to the upper end of the ‘moderate risk’ category. Nevertheless, despite the advice on discharge from the cancer family clinics, it was noted that very few of the newly occurring cancers had been reported to the breast cancer family clinics, either by the patients themselves or via the symptomatic breast services.

The practical implications of this study will not necessarily mean an increased workload for breast cancer family history surveillance programmes. While adopting current NICE rather than SIGN criteria means an increase in the proportion of referrals leading to inclusion in a surveillance programme. The actual increase is small since 60–75% of all referred women are already enrolled in special screening. Only 10% of those previously judged to be below threshold risk level (i.e., 2.5–4% of all referrals) will now be added to the surveillance programme. Although that is not a trivial consideration, the added workload (and cost) could be offset if it can be confirmed that many women currently enrolled at the age of 35 or 40 years may safely delay entry until 45 years. For at least some of those at ‘moderate’, rather than ‘high’ risk, screening from age 45 to 55 years, perhaps at intervals of 18 rather than 12 months, may prove to be cost-effective and it should be borne in mind that this risk group comprises the bulk of cancer family referrals. Hence, a reduction of some 5% in total workload should be achievable by this approach.

For the present, our findings support the NICE modification of threshold for ‘moderate risk’ – that is, removing any ‘age at diagnosis’ restriction where there are two affected close relatives. To extend our findings and to generate evidence that might justify further adjustments to family history criteria for enrolment in special surveillance programmes, we propose to continue follow-up of the cohort of women described in this report, since each additional year provides a further 2000 women years of observation and the incomplete data from 2004 onwards show that accrual of breast cancers is continuing at an undiminished rate.

Longer follow-up may also allow us to address the crucial question of whether special surveillance programmes for women with a family history of breast cancer confer any advantage in terms of outcome. It is impractical to assign such women to a randomised trial, deliberately excluding some from clinical/mammographic screening, but our cohort may generate comparable, albeit more limited, data.

Recent findings in relation to the identification of ‘low penetrance’ breast cancer susceptibility alleles (Easton *et al*, 2007; Hunter *et al*, 2007) may lead to more precise definition of individual familial risks and it will be of great interest to establish how the distribution of these alleles correlates with breast cancer incidence across a wide spectrum of risk as determined by family history.

## Figures and Tables

**Table 1a:** Data on breast cancers among 2074 women discharged from the Scottish breast cancer family clinics, January 1994 to December 2003

		**Breast cancers expected**	**Breast cancers recorded**	
**Women-years of F/U:**		**(Population data)**	**(Total cohort)**	**(Excluding NICE ‘moderate risk’)**
Age<35	843	0.25	0	0
35–39	1341	0.8	1	1
40–44	1552	1.9	2	1
45–49	1522	2.7	4	3
50–54	1405	3.9	11	8
55–59	846	2.5	5	5
60–64	454	1.4	2	0
65+	320	0.95	3	1
				
Total	8283	14.4	28	19
			(95% CI=18.61–40.47)	(95% CI=11.44–29.67)
				
	**Node negative**	**Node positive**		
Invasive ductal carcinoma	13	6		
Invasive lobular carcinoma	2	1		
Invasive tubular carcinoma	4	0		
Mixed types	2	0		
Screen-detected=18,	Symptomatic=10			

**Table 1b:** Breast cancers diagnosed in the study cohort since December, 2003

Age		
<35	0	
35–39	0	
40–44	1	(NICE ‘moderate risk’)
45–49	1	
50–54	2	
55–59	4	
60–64	3	(Two NICE ‘moderate risk’)
Total	11	7 screen-detected, 4 symptomatic: 10 invasive ductal, 1 papillary
